# Supplementary material for: Multidrug resistance plasmids underlie clonal expansions and international spread of Salmonella enterica serotype 1,4,[5],12:i:- ST34 in Southeast Asia
Source: Commun Biol. 2023 Oct 3;6:1007. doi: 10.1038/s42003-023-05365-1 (PMC10547704; doi:10.1038/s42003-023-05365-1)
Supplement: Supplementary file 3 — Description of Additional Supplementary Files [file 42003_2023_5365_MOESM3_ESM.pdf]

Description of Additional Supplementary Files

File name: Supplementary Data 1
Description: Details of Salmonella ST34 genomes used in this study.

File name: Supplementary Data 2
Description: The source data behind the graphs in this study.
