# Supplementary material for: Multidrug resistance plasmids underlie clonal expansions and international spread of Salmonella enterica serotype 1,4,[5],12:i:- ST34 in Southeast Asia
Source: Commun Biol. 2023 Oct 3;6:1007. doi: 10.1038/s42003-023-05365-1 (PMC10547704; doi:10.1038/s42003-023-05365-1)
Supplement: Supplementary file 6 — Reporting Summary [file 42003_2023_5365_MOESM6_ESM.pdf]

nature portfolio

Corresponding author(s): Hao Chung The

Last updated by author(s): 13th September 2023

Reporting Summary

Nature Portfolio wishes to improve the reproducibility of the work that we publish. This form provides structure for consistency and transparency in reporting. For further information on Nature Portfolio policies, see our Editorial Policies and the Editorial Policy Checklist.

Statistics

For all statistical analyses, confirm that the following items are present in the figure legend, table legend, main text, or Methods section.

n/a	Confirmed	
☐	☒	The exact sample size (*n*) for each experimental group/condition, given as a discrete number and unit of measurement
☒	☐	A statement on whether measurements were taken from distinct samples or whether the same sample was measured repeatedly
☐	☒	The statistical test(s) used AND whether they are one- or two-sided<br>*Only common tests should be described solely by name; describe more complex techniques in the Methods section.*
☒	☐	A description of all covariates tested
☒	☐	A description of any assumptions or corrections, such as tests of normality and adjustment for multiple comparisons
☐	☒	A full description of the statistical parameters including central tendency (e.g. means) or other basic estimates (e.g. regression coefficient) AND variation (e.g. standard deviation) or associated estimates of uncertainty (e.g. confidence intervals)
☐	☒	For null hypothesis testing, the test statistic (e.g. *F*, *t*, *r*) with confidence intervals, effect sizes, degrees of freedom and *P* value noted<br>*Give P values as exact values whenever suitable.*
☐	☒	For Bayesian analysis, information on the choice of priors and Markov chain Monte Carlo settings
☒	☐	For hierarchical and complex designs, identification of the appropriate level for tests and full reporting of outcomes
☒	☐	Estimates of effect sizes (e.g. Cohen's *d*, Pearson's *r*), indicating how they were calculated

Our web collection on statistics for biologists contains articles on many of the points above.

Software and code

Policy information about availability of computer code

Data collection	Illumina Hiseq 2500 basecalling software
Data analysis	FastQC v0.11.5, Trimmomatic v0.38, BWA-mem v0.7.17, GATK v3.7.0, bcftools v1.12, samclip, Freebayes v1.3.6, Gubbins v1.4.5, RAxML v.8.2.4, ggtree v3.2.1, Fastbaps v1.0.6, Tempest v1.5.1, BEAST v1.10.4, Tracer v.17, phytools v1.0-1, Unicycler v0.4.9, Prokka v1.14.6, Abricate v0.7, SnpEff v5.0e, PAML, Guppy v6.3.8, Easyfig, fastaq v3.17.0

For manuscripts utilizing custom algorithms or software that are central to the research but not yet described in published literature, software must be made available to editors and reviewers. We strongly encourage code deposition in a community repository (e.g. GitHub). See the Nature Portfolio guidelines for submitting code & software for further information.

Data

Policy information about availability of data

All manuscripts must include a data availability statement. This statement should provide the following information, where applicable:

- Accession codes, unique identifiers, or web links for publicly available datasets
- A description of any restrictions on data availability
- For clinical datasets or third party data, please ensure that the statement adheres to our policy

Raw sequence data are available in the European Nucleotide Archive (ENA) under the project number PRJEB9121. Full-length plasmid sequences generated in this

study are deposited in the Genbank repository under the accession numbers OQ658820- OQ658824. Supplementary Table 1 provides the detailed accession numbers and metadata for all genomes used in this study. Supplementary Data 2 provides source data for Figs 2b, 2c, 3 and 5b.

Human research participants

Policy information about studies involving human research participants and Sex and Gender in Research.

Reporting on sex and gender	Sex and gender were not relevant to this study's objective, and these information was not collected
Population characteristics	The study utilized genome sequences of Salmonella isolated from 133 Vietnamese children (age < 5) with dysenteric diarrhea
Recruitment	Participants were recruited by the study doctors at Children Hospital no. 1, Children Hospital no. 2, and Hospital for Tropical Diseases in Ho Chi Minh City, Vietnam. There is no identified self-bias in the selection.
Ethics oversight	Children Hospital no. 1, Children Hospital no. 2, Hospital for Tropical Diseases (HCMC, Vietnam), and the University of Oxford Tropical Research Ethics Committee (OxTREC no. 1045-13)

Note that full information on the approval of the study protocol must also be provided in the manuscript.

Field-specific reporting

Please select the one below that is the best fit for your research. If you are not sure, read the appropriate sections before making your selection.

☐ Life sciences ☐ Behavioural & social sciences ☒ Ecological, evolutionary & environmental sciences

For a reference copy of the document with all sections, see nature.com/documents/nr-reporting-summary-flat.pdf

Ecological, evolutionary & environmental sciences study design

All studies must disclose on these points even when the disclosure is negative.

Study description	Evolutionary history of Salmonella enterica serotype 4,[5],12,i:- ST34 in Southeast Asia
Research sample	Whole genome sequences of Salmonella enterica ST34. Existing data were compiled from Enterobase, or previously published dataset (Duong et al., JCM, 2020; Mather et al., mBio, 2018; Ingle et al., Nat Comms, 2021; Petrovska et al., EID, 2016; Arnott et al., EID, 2018; Elnekave et al., EID, 2020).
Sampling strategy	No sample size calculation was performed. We gathered all existing Salmonella ST34 genomes isolated in Vietnam, other samples were included for phylogenetic context, including (1) randomly selected sequences from other Asian countries (n=15 max), (2) phylogenetically diverse collection of genomes isolated in UK, USA and Australia. Salmonella ST34 has been estimated to emerge since early 1990s.
Data collection	Data collection was performed by the author by searching the NCBI sequence Read Archive, using the corresponding accession numbers provided by previous publications.
Timing and spatial scale	Genomes of Salmonella isolated from 2002 to 2019. The study focuses on Vietnam and Southeast Asia, with other global samples for phylogenetic context.
Data exclusions	No data were excluded in the analysis
Reproducibility	Data analysis tools, together with set parameters, were included in details in the Methods. We provided accession numbers for all genomes used in this study
Randomization	Randomization was not relevant in this study
Blinding	Blinding was not relevant in this study

Did the study involve field work? ☐ Yes ☒ No

Reporting for specific materials, systems and methods

We require information from authors about some types of materials, experimental systems and methods used in many studies. Here, indicate whether each material, system or method listed is relevant to your study. If you are not sure if a list item applies to your research, read the appropriate section before selecting a response.

Materials & experimental systems

n/a	Involved in the study
☒	☐ Antibodies
☒	☐ Eukaryotic cell lines
☒	☐ Palaeontology and archaeology
☒	☐ Animals and other organisms
☒	☐ Clinical data
☒	☐ Dual use research of concern

Methods

n/a	Involved in the study
☒	☐ ChIP-seq
☒	☐ Flow cytometry
☒	☐ MRI-based neuroimaging
